# Diagnosing sputum/smear negative pulmonary tuberculosis

**DOI:** 10.4103/0970-2113.71979

**Published:** 2010

**Authors:** Sivaraman Vengattaraman

**Affiliations:** *Tuberculosis Control Officer (Rtd), Government Chest Clinic. 68, Ambour Sabi, Pondicherry 605001, India. E-mail: vengsiva@gmaiil.com*

Sir,

I read with interest the article by Bachh *et al*.[1] I would appreciate if the following are clarified:

1. In Table 2 on P60, the total number of tuberculosis cases found is shown as 60 whereas in Table 4 (in the column exclusive diagnosis % in the row present study, it is shown as 66. I am unable to understand this difference.
2. In Table 2 (row 1), four cases which had positive culture before bronchoscopy had negative smear and culture in bronchial washing. They had non caseating granuloma and two had post bronchoscopy smear positive. This is confusing.
3. It is seen from Table 2 that 20 cases had prebronchoscopic sputum culture positive. Perhaps by waiting for 10-15 days (BACTEC culture time), the invasive procedure could have been avoided. In addition, I feel the bronchoscopist and assistant would have been exposed to the risk of infection unnecessarily. Is it justified?
